# Association of osteoporosis and skeletal muscle loss with serum type I collagen carboxyl-terminal peptide β glypeptide: A cross-sectional study in elder Chinese population

**DOI:** 10.1515/med-2023-0642

**Published:** 2023-02-15

**Authors:** Lingyan Chen, Jiayu Wu, Weiying Ren, Xi Li, Man Luo, Yu Hu

**Affiliations:** Department of Geriatrics, Zhongshan Hospital, Fudan University, Shanghai 200032, China; Department of Geriatrics, Zhongshan Hospital, Fudan University, Fenglin Road 180, Shanghai 200032, China

**Keywords:** elderly, β-CTX, skeletal muscle mass, bone mass, osteoporosis

## Abstract

Type I collagen carboxyl-terminal peptide β (β-CTX) increases in osteoporosis. The study aimed to explore the relationship between serum β-CTX and the risk of osteoporosis as well as sarcopenia in Chinese elderly inpatients. Around 228 patients whose age >65 years were recruited in this cross-sectional study. Dual-energy X-ray scanning was used to access skeletal muscle and bone mass. Serum concentration of β-CTX as well as the prevalence of osteoporosis were significantly higher in low skeletal muscle index (SMI) group than that in the normal SMI group (*P* < 0.05). Serum β-CTX levels negatively correlated with SMI and bone mass (*P* < 0.05). Total muscle mass, appendicular skeletal muscle mass, SMI, total bone mass, and bone mass at various sites including the limbs, spine, and pelvis decreased significantly, and the prevalence of low SMI increased with the increase of the quartiles of β-CTX. Higher serum β-CTX had an increased risk of low SMI and osteoporosis (*P* < 0.05). Summarily, with increasing serum β-CTX levels, both muscle and bone mass decreased in Chinese elderly inpatients. Serum β-CTX was positively associated with the risk of not only osteoporosis but also skeletal muscle loss.

## 1 Introduction

Osteoporosis is a skeletal disease characterized by low bone mineral density (BMD) and microarchitectural deterioration of bone tissue, a consequent increase in bone fragility and susceptibility to fracture [1]. It is important to note that the prevalence of osteoporosis and its related complications are expected to increase as the aging populations increase worldwide [2].

It was estimated that more than 70 million people suffered osteoporosis, and the prevalence of osteoporosis was about 20.7% in women and 14.4% in men among people aged ≥50 years in the China [3,4]. In addition to osteoporosis, sarcopenia is also an age-related disease that predominantly affects older individuals, which is characterized by progressive and generalized loss of skeletal muscle mass and strength [5]. Both osteoporosis and sarcopenia are associated with a higher risk for falls and fractures, as well as significant morbidity, disability, poor mobility, frailty, and hospitalization [6–8]. As aging has an overwhelming effect on bone and skeletal muscle, and due to the interactions between bone and skeletal muscle tissues, osteoporosis and sarcopenia often coexist in the same older individual and lead to similar consequences. Regarding the current secular trends of population aging, the burden of both diseases may continue to increase.

In addition to the similar susceptible population, there is also growing evidence on the close relationship between osteoporosis and sarcopenia [9]. It has been shown that low muscle mass or function contributed to low BMD in postmenopausal women [10,11]. Previous study considered sarcopenia as an independent risk factor for decreased BMD and osteoporosis condition among older people [12,13]. Meanwhile, patients existing with low bone mass would suffer higher risk of sarcopenia [14,15].

Since skeletal muscle and bone are closely linked, there are several potential explanations for these interrelationships. Recent studies showed that a bidirectional bone–muscle crosstalk existed, which was probably mediated by cytokines, osteokines, myokines, and other growth factors [16,17]. It was reported that cytokines from bone would affect skeletal muscle by autocrine and paracrine [17]. Meanwhile, as skeletal muscle was supposed to be the largest endocrine organ in the body, myogenic cytokines could also regulate bone structure and function [16].

The development and maintenance of bone tissue mostly depend on the coordinated actions of bone forming osteoblasts and bone absorbing osteoclasts. Most studies have presented the relationship between osteoblasts and skeletal muscle [17,18]. However, whether the activity of osteoclasts is related to sarcopenia remains unclear. Therefore, type I collagen carboxyl-terminal peptide β (β-CTX), an indicator reflecting the activity of osteoclasts, was evaluated to explore the relationship between osteoclasts and sarcopenia as well as osteoporosis in the present study.

## 2 Methods

### 2.1 Participants

Patients >65 years hospitalized in the Geriatric Department of Zhongshan Hospital, affiliated with Fudan University (Shanghai, China), from October 2017 to December 2019 were screened. All the participants had the ability of independent daily performance. Around 228 patients >65 years were recruited.

Participants were excluded as follows: (1) six patients lack of data of dual-energy X-ray scanning (DXA); (2) 42 patients suffered malignant tumors, mobility disorders, serious liver and kidney disease, and acute conditions including infection, acute stroke, acute cardiovascular disease, or surgical stress; and (3) 14 patients lack of serum data of bone metabolism. Finally, 166 participants were included in the analysis (130 males and 36 females).


**Ethics approval:** This study was approved by the Research Ethics Committee of Zhongshan Hospital affiliated to Fudan University (No. B2017-079R).
**Informed consent:** Written informed consent was obtained from all individual participants included in the study.

### 2.2 Data collection

During hospitalization, the patients’ information on lifestyle, medical history, and prior medications were collected. Weight and height were measured while the participant was clothed in a light gown. Body mass index (BMI) was calculated as weight divided by squared height (kg/m^2^).

After at least a 10 h overnight fast, a venous blood sample was collected for the examination of bone remodeling markers. Serum concentration of calcium and phosphorus was tested by colorimetry on a model 7600 automated bio-analyzer (Cobas c702, Roche, Germany). Osteocalcin, β-CTX, 25 hydroxyvitamin D [25(OH)VitD], parathyrin (PTH), and procollagen Type I N-peptide (PINP) were tested by radioimmunoassay on a model 8000 automatic immune analyzer (Cobas e801, Roche, Germany).

Body composition including lean mass, fat mass, and bone mass were measured using DXA scanner (Discovery, S/N 88576, Hologic). All measurements were carried out by a single trained technician at a single clinical center. Manual DXA analysis software was used to analyze the DXA scans. Lean mass was considered as skeletal muscle mass. Skeletal muscle mass index (SMI) was calculated as appendicular skeletal muscle mass (ASM)/height^2^. According to the criteria of Asian sarcopenia working group [10], SMI <7.0 kg/m^2^ in male, SMI <5.4 kg/m^2^ in female was considered as low SMI. Total BMD and BMD at limbs, lumbar 1–4, and femoral neck was detected. According to WHO criteria, the T-Score of BMD at lumbar 1–4 and/or femoral neck less than 1 standard deviation (SD) below peak bone value of healthy adults in the same gender and race was considered as normal, while the degree of reduction equal to or greater than 2.5 SD was considered as osteoporosis [11].

### 2.3 Statistical analysis

The sample size of this study was calculated based on the prevalence of sarcopenia whose prevalence was lower than osteoporosis, about 20% according to previous reports. All statistical analyses were performed using SPSS software version 21.0 (SPSS, Chicago, IL, USA). Continuous variables were evaluated by Shapiro–Wilk test to determine the normal distribution, and then presented as mean ± SD except for skewed variables. Categorical variables were presented as numbers and percentages. The subjects were divided into four groups according to quartiles of β-CTX as follows: *Q*1: ≦0.220 ng/mL, *Q*2: 0.221–0.310 ng/mL, *Q*3: 0.311–0.480 ng/mL, and *Q*4: ≧0.481 ng/mL. Analysis of variance was used for inter-group comparisons of continuous data and the *χ* ^2^ test was applied for the categorical variables. Pearson analysis was performed to access the relationship between β-CTX with muscle and bone mass. Multivariate logistic regression analyses were used to investigate the association between the quartiles of β-CTX and the risk of osteoporosis as well as low SMI. Odds ratio (OR) and 95% confidence intervals (CIs) of osteoporosis and low SMI in each group as compared with the reference group was calculated via the logistic regression models. For all analyses, *P* < 0.05 was considered statistically significant.

## 3 Results

### 3.1 Characteristics of the subjects

The mean age of the subjects was 82.68 ± 7.91 years old. The prevalence of osteoporosis, low SMI, and low SMI with osteoporosis was 81 (48.8%), 40 (24.1%) and 25 (15.1%), respectively. The prevalence of osteoporosis was significantly higher in low SMI group than that in the normal SMI group (62.5% vs 44.4%, *P* = 0.035). Regarding bone remodeling markers, the individuals in the low SMI group showed significantly higher β-CTX and osteocalcin compared with that in the normal group. The characteristics of the subjects are presented in Table 1.

**Table 1:** Characteristics of the subjects

	Total (*n* = 166)	Low SMI (*n* = 40)	Normal (*n* = 126)	*P*-value
Male (*n*,%)	130, 78.3%	29, 72.5%	101, 80.2%	0.209
Age (years)	82.68 ± 7.91	86.02 ± 6.78	81.49 ± 8.76	0.002
BMI (kg/m^2^)	24.23 ± 3.20	21.18 ± 1.86	24.98 ± 2.93	<0.001
TSM (kg)	52.34 ± 9.23	43.58 ± 7.21	53.78 ± 8.58	<0.001
ASM (kg)	20.91 ± 4.07	16.76 ± 3.15	21.63 ± 3.74	<0.001
SMI (kg/m^2^)	7.46 ± 1.04	6.12 ± 0.64	7.74 ± 0.87	<0.001
TBM (kg)	2.21 ± 0.44	1.92 ± 0.39	2.26 ± 0.44	<0.001
TBMD (g/cm^2^)	1.11 ± 0.13	1.05 ± 1.21	1.12 ± 1.28	0.001
ABM (kg)	1.17 ± 0.27	0.97 ± 0.22	1.21 ± 0.27	<0.001
ABMD (g/cm^2^)	0.98 ± 0.12	0.89 ± 0.11	0.99 ± 0.12	<0.001
SBM (kg)	0.19 ± 0.05	0.16 ± 0.05	0.20 ± 0.06	0.003
SBMD (g/cm^2^)	1.01 ± 0.20	0.92 ± 0.16	1.03 ± 0.21	0.006
PBM (kg)	0.21 ± 0.06	0.17 ± 0.05	0.22 ± 0.07	<0.001
PBMD (g/cm^2^)	1.15 ± 0.20	1.03 ± 0.16	1.18 ± 0.20	<0.001
Osteoporosis (*n*,%)	81, 48.8%	25, 62.5%	56, 44.4%	0.035
Calcium (mmol/L)	2.23 ± 0.11	2.22 ± 0.10	2.24 ± 0.11	0.588
Phosphorus (mmol/L)	1.07 ± 0.15	1.10 ± 0.15	1.06 ± 0.16	0.159
Osteocalcin (ng/mL)	14.63 ± 7.82	18.47 ± 11.04	13.60 ± 6.92	0.001
β-CTX (ng/mL)	0.39 ± 0.23	0.52 ± 0.35	0.34 ± 0.19	<0.001
PINP (ng/mL)	47.79 ± 28.00	54.55 ± 27.94	44.94 ± 27.75	0.059
25(OH)Vit D (nmol/L)	48.12 ± 25.81	52.89 ± 35.80	48.43 ± 23.69	0.365
PTH (pg/mL)	44.17 ± 18.33	46.03 ± 20.19	42.93 ± 17.29	0.349
ALP (U/L)	65.61 ± 25.97	64.57 ± 15.63	65.31 ± 28.26	0.876

TSM: total muscle mass; ASM: appendicular muscle mass; FM: fat mass; FM%: percentage of fat mass; TBM: total bone mass; TBMD: total bone mineral density; LBM: appendicular bone mass; ABMD: appendicular bone mineral density; SBM: spinal bone mass; SBMD: spinal bone mineral density; PBM: pelvis bone mass; PBMD: pelvis bone mineral density; β-CTX: Type I collagen carboxyl-terminal peptide β; 25(OH)VitD: 25 hydroxyvitamin D; PTH: parathyrin; PINP: procollagen Type I N-peptide; ALP: alkaline phosphatase.

### 3.2 Association of β-CTX with skeletal muscle mass and bone mass

Pearson analysis showed that serum β-CTX levels were significantly positively associated with age, osteocalcin, PINP, and PTH (all *P* < 0.05). Regarding the body compositions, β-CTX was significantly negatively related with BMI, SMI, total skeletal muscle mass (TSM), appendicular skeletal muscle mass (ASM), total bone mass (TBM), appendicular bone mass (ABM), and pelvic bone mass (PBM) as well as BMD at the corresponding sites (all *P* < 0.05) (as shown in Table 2).

**Table 2:** Pearson analysis of the association of β-CTX and clinical characteristics

Age	0.18, 0.017
BMI	−0.24, 0.002
Ca	−0.09, 0.247
Calcium	0.07, 0.370
Phosphorus	0.05, 0.479
Osteocalcin	0.85, <0.001
PINP	0.65, <0.001
25Vit D	−0.08, 0.306
PTH	0.34, <0.001
TSM	−0.23, 0.003
ASM	−0.25, 0.001
SMI	−0.34, <0.001
TBM	−0.23, 0.003
TBMD	−0.20, 0.009
ABM	−0.26, 0.001
ABMD	−0.28, <0.001
SBM	−0.15, 0.053
SBMD	−0.17, 0.032
PBM	−0.28, <0.001
PBMD	−0.32, <0.001

**Figure 1:**
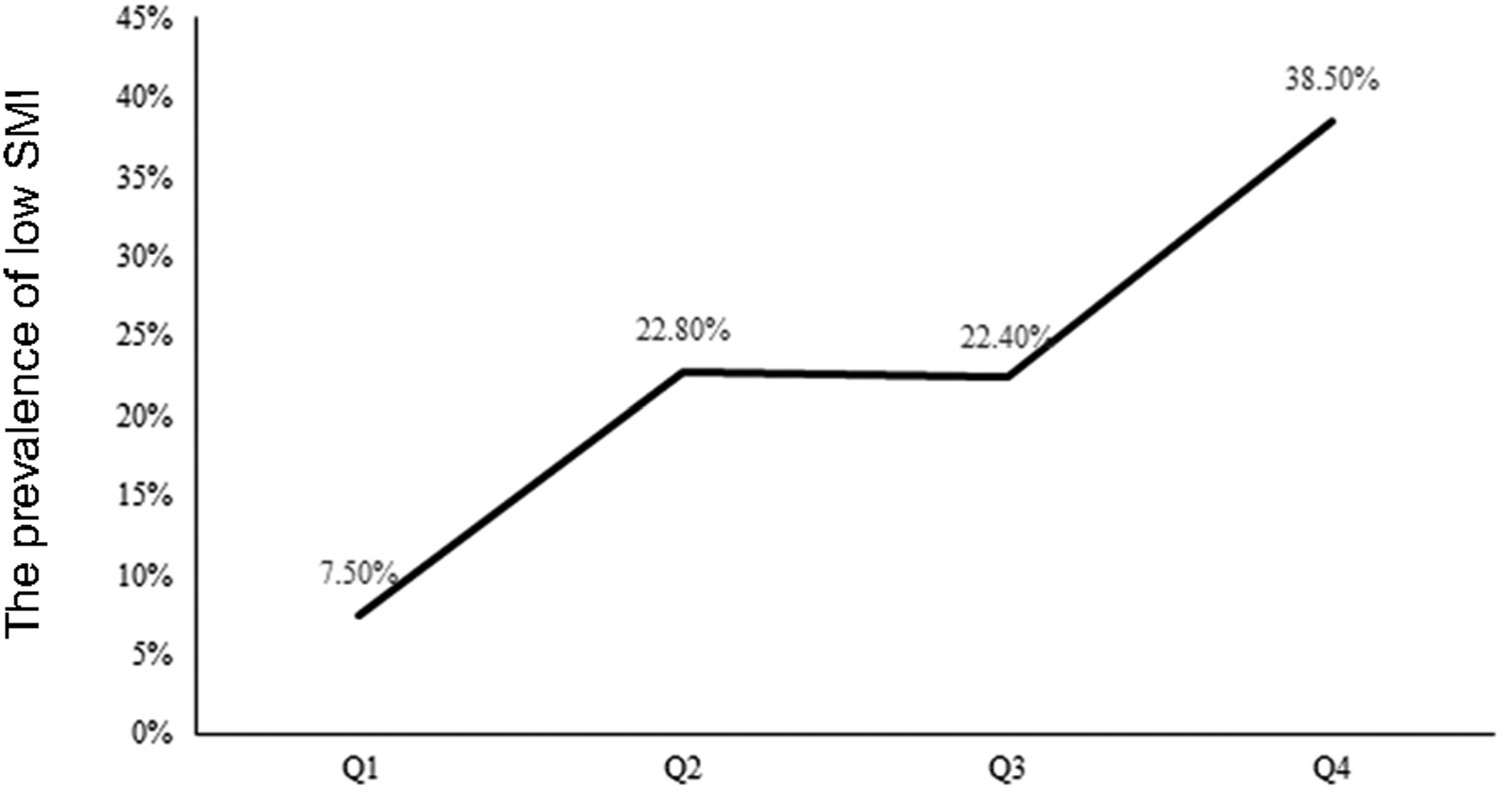
Prevalence of low SMI across the quartiles of serum β-CTX. The quartiles of serum β-CTX: *Q*1: ≦0.220 ng/mL, *Q*2: 0.221–0.310 ng/mL, *Q*3: 0.311–0.480 ng/mL, and *Q*4: >0.480 ng/mL.

To further explore the association between β-CTX and skeletal muscle and bone mass, participants were stratified by the β-CTX quartiles. Compared with the participants in the lowest β-CTX quartile, the participants in the higher quartiles were older and presented with lower BMI. In accordance with the increase of the quartiles of β-CTX, SMI, TSM, ASM, TBM, ABM, spinal bone mass (SBM), and PBM as well as BMD all decreased. The prevalence of low SMI accordingly increased, presented as 7.5, 22.8, 22.4, and 38.5%, respectively (Figure 1). The index of bone formation including osteocalcin, PTH, and PINP increased across the quartiles. The results are presented in Table 3.

**Table 3:** Characteristics of the subjects according to the quartiles of serum β-CTX

	*Q*1	*Q*2	*Q*3	*Q*4	*P*-value
	*Q* = 46	*Q* = 37	*Q* = 42	*Q* = 41	
Age (years)	80.65 ± 7.92	82.19 ± 7.49	83.78 ± 7.35	84.15 ± 8.58	0.147
BMI (kg/m^2^)	24.75 ± 3.48	24.05 ± 3.37	24.39 ± 2.80	23.15 ± 2.94	0.121
Calcium (mmol/L)	2.26 ± 0.12	2.21 ± 0.09	2.21 ± 0.11	2.23 ± 0.11	0.106
Phosphorus(mmol/L)	1.07 ± 0.16	1.06 ± 0.16	1.02 ± 0.17	1.11 ± 0.12	0.054
Osteocalcin (ng/mL)	8.98 ± 2.93	12.15 ± 3.97	14.25 ± 4.26	23.09 ± 9.08	<0.001
PINP (ng/mL)	28.59 ± 9.08	41.64 ± 15.73	49.21 ± 32.39	70.16 ± 28.16	<0.001
25Vit D (nmol/L)	48.79 ± 24.79	44.21 ± 24.88	58.70 ± 29.73	46.33 ± 27.11	0.075
PTH (pg/mL)	34.98 ± 17.17	43.79 ± 16.25	44.25 ± 12.14	53.47 ± 21.13	<0.001
ALP (U/L)	61.78 ± 40.97	61.57 ± 15.48	65.07 ± 16.68	71.02 ± 16.95	0.306
TSM (kg)	54.07 ± 9.69	52.24 ± 8.82	52.18 ± 8.57	47.94 ± 9.65	0.023
ASM (kg)	21.83 ± 4.33	21.05 ± 3.67	20.63 ± 3.83	19.02 ± 4.21	0.015
SMI (kg/m^2^)	7.82 ± 1.14	7.52 ± 1.01	7.33 ± 0.93	6.92 ± 0.96	0.001
TBM (kg)	2.27 ± 0.47	2.31 ± 0.44	2.16 ± 0.37	1.97 ± 0.46	0.003
TBMD (g/cm^2^)	1.13 ± 0.14	1.15 ± 0.12	1.09 ± 0.11	1.05 ± 0.13	0.003
ABM (kg)	1.22 ± 0.26	1.21 ± 0.25	1.15 ± 0.25	1.03 ± 0.30	0.003
ABMD (g/cm^2^)	1.01 ± 0.12	1.00 ± 0.12	0.95 ± 0.11	0.91 ± 0.13	0.001
SBM (kg)	0.19 ± 0.06	0.20 ± 0.06	0.19 ± 0.05	0.16 ± 0.05	0.026
SBMD (g/cm^2^)	1.04 ± 0.19	1.05 ± 0.23	0.99 ± 0.21	0.92 ± 0.16	0.023
PBM (kg)	0.23 ± 0.08	0.22 ± 0.07	0.20 ± 0.05	0.17 ± 0.05	0.001
PBMD (g/cm^2^)	1.22 ± 0.24	1.17 ± 0.19	1.13 ± 0.17	1.04 ± 0.15	0.001

The quartiles of serum β-CTX: *Q*1: ≦0.220 ng/mL, *Q*2: 0.221–0.310 ng/mL, *Q*3: 0.311–0.480 ng/mL, and *Q*4: >0.480 ng/mL.

### 3.3 Logistic regression analysis of the relationship between β-CTX and osteoporosis as well as low SMI

Logistic regression analysis was conducted to verify the relationship between serum β-CTX and the risk of osteoporosis as well as low SMI. The results showed that higher levels of serum β-CTX were associated with increased risk of skeletal muscle loss and osteoporosis (*P* < 0.019). Compared to the lowest quartile of serum β-CTX, after multiple adjustments, the third and fourth quartiles of serum β-CTX had an increased risk of low SMI (the third quartile: OR = 5.84, 95%CI = 1.21–28.34, *P* = 0.028; the fourth quartile: OR = 6.80, 95%CI = 1.38–33.60, *P* = 0.019) and the fourth quartile of serum β-CTX had an increased risk of osteoporosis (OR = 3.39, 95%CI = 1.20–9.55, *P* = 0.021) (Table 4).

**Table 4:** Logistic analysis of the relationship between quartile of serum β-CTX and osteoporosis as well as low SMI

	*Q*1	*Q*2	*Q*3	*Q*4
**Osteoporosis**				
Unadjusted	1	3.18 (1.20–8.42, 0.020)	1.92 (0.74–4.97, 0.179)	6.75 (2.52–18.12, <0.001)
Model 1	1	3.11 (1.16–8.33, 0.024)	1.63 (0.61–4.35, 0.325)	5.18 (1.86–14.45, 0.002)
Model 2	1	2.91 (1.06–7.98, 0.038)	1.55 (0.57–4.19, 0.390)	4.36 (1.53–12.43, 0.006)
Model 3	1	2.89 (1.03–8.11, 0.044)	1.61 (0.58–4.48, 0.357)	4.41 (1.51–12.93, 0.007)
Model 4	1	2.63 (0.96–7.17, 0.060)	1.39 (0.51–3.73, 0.520)	3.39 (1.20–9.55, 0.021)
**Low SMI**				
Unadjusted	1	3.65 (0.89–15.61, 0.073)	3.98 (1.01–15.75, 0.049)	7.71 (2.01–29.49, 0.003)
Model 1	1	3.41 (0.82–14.29, 0.093)	3.45 (0.85–14.03, 0.083)	6.41 (1.59–25.83, 0.009)
Model 2	1	3.09 (0.53–17.86, 0.207)	6.29 (1.06–37.42, 0.043)	6.97 (1.16–41.90, 0.034)
Model 3	1	2.77 (0.49–15.67, 0.249)	5.79 (1.02–32.89, 0.047)	6.44 (1.10–37.61, 0.039)
Model 4	1	2.83 (0.59–13.49, 0.192)	5.84 (1.21–28.34, 0.028)	6.80 (1.38–33.60, 0.019)

Model 1: gender, age.

Model 2: gender, age, BMI.

Model 3: gender, age, BMI, VitD medication.

Model 4: gender, age, BMI, VitD medication, low SMI (for osteoporosis); gender, age, BMI, VitD medication, osteoporosis (for low SMI).

## 4 Discussion

In the present study, the elderly inpatients who suffered low SMI had lower bone mass and BMD, while the prevalence of osteoporosis was higher. The results indicated close association between skeletal muscle loss and osteoporosis, which was similar with previous studies [9–11]. Not only BMD but also bone mass reduction were associated with muscle loss, which further supported the association between sarcopenia and osteoporosis. As skeletal muscle and bone are the two major components of the musculoskeletal system, they have a close mechanical relationship as well as biochemical crosstalk [17]. Bone provides attachment sites for muscle, and skeletal muscle imparts a force on the bone to facilitate locomotion of the organism. In addition, muscle-derived force is also the main source of mechanical loads that generate the strain in the bone. It has been proposed that several cytokines were involved in muscle and bone biochemical crosstalk, such as IGF-1, Irisin, FGF-23, IL-6, and sclerostin [19]. Besides, some well-known genes are associated with muscle loss and osteoporosis concurrently, for example myostatin, proliferator-activated receptor gamma coactivator 1-α, myocyte enhancer factor-2 C, and methyltransferase-like 21C [20].

Osteoblasts and osteoclasts are the main factors involved in bone remodeling in the bone micro-environment. As the degradation products of type I collagen, the serum concentration of β-CTX reflects bone turnover activity, and the relationship between β-CTX and osteoporosis has been well confirmed [21]. Patients with osteoporosis often presented with elevated β-CTX, which represented the increase of the activity of bone resorption [22]. The present study showed that with the increase of β-CTX, both bone mass and BMD at corresponding sites decreased, which indicated that the activity of osteoclasts affected not only bone contents but also bone microstructure. With the increase of the serum β-CTX, bone formation markers such as PINP and osteocalcin increased simultaneously. This indicated that the function of osteoblasts might compensatory enhance with the increased activity of osteoclast.

The linkage of osteoblasts and muscle has been investigated [23,24]. However, whether the action of osteoclasts was involved in the association between osteoporosis and sarcopenia was still unclear. Our study first revealed that elevated serum β-CTX was not only associated with osteoporosis but also skeletal muscle loss. With the increase of serum β-CTX, both bone and skeletal muscle mass decreased. Quartile analysis added further evidence in that there was a positive linear correlation between serum β-CTX and the risk of low SMI in Chinese elderly inpatients. Compared to the lowest quartile of serum β-CTX, patients with β-CTX greater than 0.311 ng/mL had a 4.8-fold increasing risk of low SMI, and HR could go up to 6.80 while β-CTX greater than 0.480 ng/mL. This indicated that the activation of osteoclasts might participate in muscle mass loss through some unknown pathways. Previous study has reported that extracellular vesicles secreted from mouse muscle might be a crucial mediator of muscle–bone interactions, which suppressed osteoclast formation by receptor activator of nuclear factor κB ligand (RANKL) [25]. Myostatin, a negative myokine of both muscle and bone through activating receptor type IIB-mediated TGF-β-specific Smad2/3 signaling, could affect the osteoclastogenesis and subperiosteal resorption [26,27]. Other skeletal-muscle secreted myokines such as irisin, could increase bone resorption on several substrates *in situ* [28]. All these evidence support the close association between muscle atrophy and osteoclasts action. But so far there was no evidence proving that osteoclasts were directly involved in the changes of muscle mass. Moreover, most previous research studies were performed in animal models, and the relationship between osteoclasts and muscle loss in humans had not been reported. The present study first proved that serum β-CTX in elderly inpatients was positively associated with the risk of skeletal muscle mass, which provided clues to the viewpoint that increasing osteoclasts activity might contribute to skeletal muscle loss, and these would indicate directions for future research. Our results revealed that besides osteoporosis, osteoclast-derived factors might also be potential therapeutic targets for sarcopenia.

The present study first explored the association between serum β-CTX and skeletal muscle mass in Chinese elderly inpatients and revealed the close association between osteoclasts activity and muscle loss, which indicated that increasing activity of osteoclasts might contribute to the skeletal muscle loss. The results provided a new possible direction for the treatment of sarcopenia. There were also some limitations in this study. First, the sample size of this study was relatively small, which could have affected the strength and significance of associations between osteoclasts and skeletal muscle mass. Second, it was a cross-sectional study, and the results needed to be further evaluated in longitudinal studies. Third, considering that current sarcopenia definitions specify the presence of low muscle strength or physical function, the information of physical activity, handgrip strength, or gait speed are still needed in further studies. Studies on the mechanism of the crosstalk between muscle and osteoclasts are needed in further study to provide a deep understanding of the association between β-CTX and muscle loss.

The study has some limitations. This study consisted of patients aged 65 years old and older, with few female participants. In addition, it included only hospitalized patients. Therefore, the sample is not representative, especially for the female population.

In conclusion, our results first demonstrated a significant correlation between serum β-CTX and skeletal muscle mass. The elevation of serum β-CTX was positively associated with the risk of muscle loss in elderly Chinese inpatients. Osteoclasts activity may be involved in the development of sarcopenia. Further prospective studies and animal models on the causal effect of osteoclasts activity on skeletal muscle reduction were required, which might not only expand our understanding on the mechanism of the relationship between osteoclasts and muscle, but also assist in the development of new prevention strategies for sarcopenia.
